# Fermentable fiber supplementation in pigs promotes anti-parasitic defense mechanisms and impacts parasite growth in *Ascaris suum* infection

**DOI:** 10.3389/fimmu.2026.1788681

**Published:** 2026-07-22

**Authors:** Philipp Höfler, Josephine Schlosser-Brandenburg, Arkadi Kundik, Sebastian Rausch, Eva-Maria Saliu, Larissa Oser, Anja A. Kühl, Robert M. Mugo, Alexandra Laubschat, Zaneta Musimbi Kidiavai, Ankur Midha, Robert Klopfleisch, Jürgen Zentek, Susanne Hartmann

**Affiliations:** 1 Department of Veterinary Medicine, Institute of Immunology, Centre for Infection Medicine, Freie Universität Berlin, Berlin, Germany; 2 Methodology and Research Infrastructure, Animal Experimental Research and 3R (MF3), Robert-Koch-Institute, Berlin, Germany; 3 Department for Landscape, Society and Economy, University of Applied Science for Sustainable Development, Eberswalde, Germany; 4 Charité Universitätsmedizin Berlin, Corporate Member of Freie Universität Berlin and Humboldt-Universität zu Berlin, iPATH.Berlin, Core unit of Charité, Campus Benjamin Franklin, Berlin, Germany; 5 Kenya Medical Research Institute (KEMRI), KEMRI-Wellcome Trust, Kilifi, Kenya; 6 Department of Veterinary, Institute of Veterinary Pathology, Freie Universität Berlin, Berlin, Germany; 7 Department of Veterinary Medicine, Institute of Animal Nutrition, Freie Universität Berlin, Berlin, Germany

**Keywords:** *Ascaris suum*, dietary fiber, helminth infection, pig, porcine models

## Abstract

Ascarids are among the most prevalent soil-transmitted helminths affecting both humans and livestock, particularly pigs. While reduced anthelmintic efficacy has been reported in humans, frequent reinfection and the lack of a vaccine highlight the need for alternative control strategies across species. In pigs, fermentable dietary fibers have been shown to enhance type 2 immune responses and mucosal barrier function and may represent a complementary strategy for parasite control. Here, we investigated the effects of a fermentable fiber diet in pigs infected with the parasite *Ascaris suum (A. suum)*. Weaned pigs were fed either a diet enriched with fermentable fibers (HFD) or a control diet low in fermentable fibers (LFD). Four weeks after initiating supplementation, pigs were infected with *A. suum* eggs and maintained on the respective diets for an additional five weeks. HFD supplementation did not affect worm burden but significantly reduced worm size. This was associated with enhanced systemic and mucosal type 2 immune responses. Small intestinal Th2 responses, goblet cell expansion and the production of the anti-helminth effector molecules Arg1 and RELM-β were increased, along with elevated peripheral eosinophil counts. Hence, dietary supplementation with HFD promoted innate and adaptive Th2 responses in *A. suum* infected pigs leading to impaired parasite development. These findings suggest that fermentable dietary fibers such as inulin and sugar beet pulp can influence infection dynamics at both the host and parasite levels.

## 1 Introduction

*Ascaris suum* (*A. suum*) is an intestinal parasite of domestic pigs and widespread in herds worldwide. Although infestations are often subclinical, they can cause a range of clinical and pathological effects. Gastrointestinal signs include diarrhea, while systemic effects such as reduced growth performance and decreased feed intake are frequently observed (1). Hepatic lesions typically manifest as multifocal interstitial hepatitis, so called “milk spots” (2, 3). During larval migration through the lungs, young pigs may develop respiratory distress characterized by pronounced expiratory effort and coughing, which can even cause death in severe cases. Infections can also lead to pneumonia, pleurisy, or allergic asthma (4, 5). Occasionally, aberrant migration results in unusual complications such as bile duct obstruction or intestinal wall perforation (6, 7). These resulting health issues consequently contribute to financial losses in pig production (8–10). Based on a postulated liver condemnation rate of 19% and an average liver price of €2.46 (11), the annual economic loss for Germany, Europe’s largest pig producer in 2023, was estimated to be approximately €20.5 million (12).

Ascarids are the most frequently detected nematodes of pigs in outdoor farming systems (13, 14). In Germany, environmental contamination was detected in 82% of the examined outdoor-reared pig farms (15). Similarly, on Dutch farms, *A. suum* was detected on 72.7% of organic units and 50% of free-range holdings (13). These high infection rates are closely linked to the parasite’s environmental stage: *A. suum* eggs get shed millionfold with the feces of even one infected animal. They are highly resilient and can remain viable in soil for up to nine years (16).

The life cycle of *Ascaris* is complex and involves migration through host tissues (17). After ingestion of infective eggs, larvae hatch in the small intestine, penetrate the large intestinal mucosa, enter the portal circulation and migrate through the liver and lungs before returning to the gut to mature (18). The hepatic phase reflects the transit through the liver accompanied by the occurrence of milk-spot lesions and during the pulmonary phase, capillaries in the lung are getting damaged, predisposing the host to secondary infections (5, 19, 20).

Helminths such as *A. suum* induce a type 2 immune response characterized by T helper 2 (Th2) cells and the cytokines interleukin (IL)-4, IL-5, and IL-13 (21, 22). These cytokines induce eosinophil recruitment, goblet cell hyperplasia, and mucus hypersecretion, all of which have the potential to interfere with worm establishment. They also augment tissue repair processes that help to restrict damage induced by migrating larvae (23). In pigs, adult *A. suum* infection is associated with impaired growth performance (1, 8). Consistent with this, adult infection reduces intestinal glucose, amino acid, and peptide transport (24). Moreover, concurrent *A. suum* infection lowers the efficacy of *Mycoplasma hyopneumoniae* vaccination administered during the finishing period (25).

Although anthelmintics against roundworms have proven effective, the vast output of eggs by adult females (over 200,000 eggs per female adult worm per day (26)) and their extreme longevity in the environment lead to rapid reinfection (15, 27). In this context emerging concerns about drug resistance are undeniable. Regarding the human counterpart *Ascaris lumbricoides* (*A. lumbricoides*), reduced cure rates of up to 90% have been observed in schoolchildren in Rwanda after treatment with the commonly used anthelmintic drug albendazole (28–31). In addition, no licensed vaccine is currently available (32, 33), emphasizing the need for complementary and alternative control measures (34). Furthermore, the demand for organic pork is rising in many countries in Europe (35, 36) and the European Union intends to increase the share of organic agriculture to 25% by 2030 (37). This trend underscores the need for non-chemical control strategies, especially given consumer preferences.

One promising avenue is nutritional modulation, particularly through dietary fiber.

In non-ruminant mammals, dietary fibers are a heterogeneous group of plant-derived carbohydrates that resist host digestion but differ fundamentally in their fermentability. This determines how they are utilized by the gut microbiota and how they influence host parasite interactions. Fermentable fibers include mostly soluble non-starch polysaccharides (NSP) such as pectin and hemicelluloses (e.g. found in plants like sugar beet (38)), or inulin from chicory root. They are fermented by intestinal microbes, producing short-chain fatty acids (SCFAs). These microbial metabolites modulate the epithelial barrier, mucin formation, and immune activity, thereby shaping the intestinal environment and immune tone (39–41). In contrast, non- or poorly fermentable fibers such as e.g. cellulose, lignin and lignocellulose resist microbial degradation and mainly increase digesta bulk and passage rate, with rather limited influence on microbial metabolism or production of immune-active metabolites like SCFAs (41, 42). In the current study, we investigated the impact of fermentable fibers on the host immune response to roundworm infection.

Previous comparative studies (43) explored dietary interventions in *A. suum* infection trials with diets containing both fermentable and non-fermentable fibers but focused primarily on the feed processing effect. The comparative effects of dietary fibers, including fermentable and non-fermentable sources, on helminth infections and host immunity remain poorly characterized. However, fermentable fiber enriched diets have been applied in the hindgut residing nematode *Trichuris suis* (*T.* suis) and were found to enhance microbial metabolite production and mucosal Th2-type immunity (44, 45). Moreover, in a trickled coinfection with *T. suis* and *A. suum*, inulin led to a 64% reduction in *A. suum* worm burden 6 weeks after the first infection dose in weaned pigs fed an inulin-rich chicory-root diet, showing that highly fermentable fibers can markedly influence nematode establishment and host immunity (46).

The present study investigates whether and how supplementation with highly fermentable fibers modify the host intestinal immunological milieu to enhance an anti-parasitic immune response. By using a well-defined dietary intervention and a moderate infection dose (4,000 *A. suum* eggs), we elucidated how increased fiber fermentability influences parasite establishmentand host innate and adaptive Th2 responses at the site of infection as well as systemically in pigs.

## 2 Materials and methods

### 2.1 Ethics statement

This animal experiment was performed at Freie Universität Berlin under ethical approval from the Berlin State Office of Health and Social Affairs (Landesamt für Gesundheit und Soziales, approval number G0107/22). The experiment is aligned with German animal welfare law and the European Convention for the Protection of Vertebrate Animals Used for Experimental and Other Scientific Purposes.

### 2.2 Generation of infective *A. suum* eggs

Infective *A. suum* eggs were processed as described previously (47). In brief, female adult *A. suum* worms were collected at a local slaughterhouse and cultured overnight in Hank’s Balanced Salt Solution (HBSS) supplemented with 200 U/ml penicillin, 200 μg/ml streptomycin, 50 μg/ml gentamycin, and 2.5 μg/ml amphotericin B (all from PAN-Biotech GmbH, Aidenbach, Germany) at 37 °C and 5% CO_2_. Eggs were harvested from the culture supernatant, washed multiple times in HBSS, transferred to distilled water with 0.1% formalin, and incubated in the dark for 6–8 weeks at 33 °C for embryonation. Three washing steps with distilled water, removal of the uterine layer, and then incubating the eggs at 37 °C in 5% HClO for 5 min, were followed by five steps of washing with distilled water and 5% bovine serum albumin (BSA, PAN-Biotech GmbH, Aidenbach, Germany). Later, eggs were purified using a sucrose density gradient with an underlying step gradient technique (fully described in (47)). The fully embryonated eggs were recovered, washed, and resuspended in HBSS Solution. The batch applied in the infection trial displayed an embryonation rate of 98.8% as determined by microscopy.

### 2.3 Pig trial

Twenty-four female German hybrid pigs (offspring of Pietrain sire crossed with female hybrids of German Landrace x Large White) from a conventional breeding facility (Brandenburg, Germany) were used at an age of 7 weeks and a weight of 13.0 kg ± 1.27 (mean and SD). To control for sex-related variability in immune responses, only female pigs were included in this study. This decision was based on well-documented evidence that biological sex significantly influences susceptibility, immune response, and disease severity in parasitic infections, largely due to hormonal and physiological differences (48–50).

The pigs were divided into four experimental groups, housed in six separate rooms (four animals per room). Half of the animals were assigned to a diet enriched in fermentable fiber, consisting of 5% inulin (Orafti HP; Beneo GmbH, Mannheim, Germany) and 5% ground sugar beet pulp (all other dietary components were obtained from Lindenberger Mühle, Ahrensfelde, Germany), later increased to 8% fermentable fiber (hereafter referred to as the high fermentable fiber diet, HFD). The remaining animals received a control diet low in fermentable fiber (LFD), in which fermentable fiber sources were replaced by non-fermentable lignocellulose. For each dietary group, a total of 12 animals were used. Of these, eight animals (divided equally between two rooms, with four animals per room) were infected with an inoculum containing 4,000 embryonated *Ascaris suum* eggs. The remaining four animals in each dietary group were housed together in a single room, left uninfected, and served as the control group for comparison.

Two weeks post arrival, coproscopy was performed on fecal samples of all pigs using the Mini-FLOTAC method (Unit of Parasitology and Parasitic Diseases at the University of Naples Federico II) (51) as explained in detail in 4. Weekly fecal samples were collected to monitor egg shedding, alongside assessments of general health and fecal consistency.

Blood sampling was conducted using a maxillary sling, a single-use 7 cm cannula (Ehrhardt Medizinprodukte GmbH, Geislingen, Germany), and an EDTA Monovette with Luer adapter (Sarstedt AG & Co. KG, Nümbrecht, Germany). A blood sample was collected via the jugular vein one day before infection. Another blood sample was collected under anesthesia via heart puncture prior to euthanasia.

Pigs were under deep general anesthesia induced by intramuscular injection of azaperone (2 mg/kg body weight; Stresnil^®^, Janssen-Cilag GmbH, Germany), ketamine hydrochloride (33 mg/kg; Ursotamin^®^, Serumwerk Bernburg AG, Bernburg, Germany), and xylazine (6 mg/kg), administered 15–30 minutes prior to euthanasia. Complete unconsciousness was verified by checking for the absence of ocular and nasal septum reflexes. Euthanasia was performed by intracardiac injection of T61 (10 mg/kg; containing tetracaine hydrochloride, mebezonium iodide, and embutramide; Intervet Deutschland GmbH, Unterschleißheim, Germany). The syringe remained in the heart to detect heartbeat. Final cardiac arrest was confirmed by cardiac auscultation with a stethoscope aligning EU directive 2010/63/UE (Annexe IV).

During the trial, two pigs from the “*Ascaris* + HFD” group were euthanized for reasons unrelated to the experimental procedures. One pig was euthanized due to a cardiogenic shock occurring during blood collection caused by an anatomical abnormality which was confirmed by a trained veterinary pathologist. The other pig was euthanized due to rectal prolapse – a disease condition which has the highest incidence in young pig between 7 and 16 weeks of up to 15% (52), which was initially treated surgically using a purse-string suture. However, recurrence occurred the following day, and after further disruption of the suture by other pigs, the decision was made to euthanize the animal for animal welfare reasons.

### 2.4 Quantification of parasitic eggs and morphological characterization of juvenile worms

To measure the occurrence of *A. suum* eggs in feces, the Mini-FLOTAC method was used as previously described in (51). 5 g of fecal material pooled from all pigs housed within the same room was homogenized in saturated saline solution. After passing the mixture through a 500 µm sieve, the filtrate was examined microscopically using two Mini-FLOTAC chambers per sample, in accordance with the manufacturer’s instructions. All pre-mature worms were collected at the dissection from the intestines of *A. suum* infected pigs at 5 weeks post-infection, counted, and immediately incubated in warm HBSS without glucose, supplemented with 200 U/ml penicillin, 200 μg/ml streptomycin, 2.5 μg/ml amphotericin, and 50 μg/ml gentamycin (all antibiotics derive from PAN-Biotech GmbH) before being processed as described in the next section. Juvenile worms were photographed on a black, non-reflective surface to ensure optimal contrast, with the camera positioned at a consistent distance and using standardized image capture settings. A regular ruler was included in each image as a scale reference, facilitating the conversion of pixel measurements into physical lengths. The images were processed using an in-house implemented Fiji-based workflow (53) (software version 2.9.0) for the standardized measurement of *A. suum* morphology. Each 8-bit image was then converted to a binary (black and white) image by applying a thresholding method, ensuring the worms were clearly distinguished from the background. Worm area were measured using the ‘Analyze Skeleton’ plugin in Fiji (54), while worm length was determined by first altering the worm images using the ‘Skeletonize’ plugin on the binary images.

### 2.5 Preparation of excretory and secretory products from juvenile *Ascaris suum*

Cultured motile female and male worms from the pig trial were separated and washed in 0.9% NaCl solution pre-incubated overnight at 37 °C. These were then cultured at a density of 1g of juvenile worms per 20 ml of medium for 24h at 37 °C in culture solution consisting of HBSS without glucose, supplemented with 200 U/ml penicillin, 200 μg/ml streptomycin, 2.5 μg/ml amphotericin and 50 μg/ml gentamycin (all from PAN Biotech). Worms were then transferred to fresh culture solution, at the same density, and incubated for a further 24h. Following the removal of worms, the culture solution from the second 24-hour incubation was stored at 4 °C overnight to allow sedimentation of particulates. The supernatant was removed and centrifuged at 10,000 x g to pellet further particulate matter. The resulting supernatant was filtered through a 450 nm filter. The filtrate was then concentrated using Vivaspin columns with a 5kDa molecular weight cut-off (Sartorius; Göttingen, Germany), rebuffered with phosphate-buffered saline (PBS), and subsequently passed through a 200 nm filter. Protein concentration was determined using the bicinchoninic acid (BCA) assay (ThermoFisher, Waltham, USA) according to an established protocol (55) and the excretory/secretory (E/S) products were diluted in PBS to a stock concentration of 1 mg/ml. Lipopolysaccharide (LPS) content was assessed using the Limulus Amoebocyte Lysate (LAL) assay (Lonza; Basel, Switzerland), with an LPS concentration measured at 1 μg/mL in the stock solution. E/S products pooled from 60 juvenile worms were utilized for the experiments.

### 2.6 Leukocyte isolation

Single cell suspensions from lymph nodes were prepared after removing fat and connective tissue. Cells from small intestinal and a pool of cecum and colon lymph nodes were isolated separately. The tissues were cut into small pieces and passed through a 70µm cell strainer using a 5mL syringe plunger (Braun, Melsungen, Germany). The cells were centrifuged, washed, and treated with erythrocyte lysing solution (0.01M KHCO_3_, 0.155M NH_4_Cl, 0.1mM EDTA, pH 7.5) to lyse residual erythrocytes for 5 minutes at room temperature. Lysis was stopped with complete Iscove’s modified Dulbecco’s medium supplementated with 100U/mL penicillin, 100μg/mL streptomycin, and 10% fetal calf serum (FCS) (cIMDM; all from PAN Biotech GmBH). The cells were centrifuged, washed again, resuspended in cIMDM, and counted using a CASY Cell Counter & Analyzer (OMNI Life Science GmbH & Co. KG, Bremen, Germany). Cell suspensions of all organs were adjusted to a concentration of 2×10^7^cells/mL for further use.

Small and large intestine samples (5×5cm) were cleaned by removing fat, muscle, connective tissue, and satellite patches, if applicable. The intestinal tissues were washed thoroughly in Ca/Mg^2+-^free HBSS that contained 10mM HEPES, 16.6mM NaHCO3, and 2% FCS (all Carl Roth GmbH & Co.KG, Karlsruhe, Germany) to remove fecal material and cut into 1×1cm pieces. These pieces were transferred to 50mL conical tubes (Greiner, Kremsmünster, Austria) containing 40mL of medium at 4 °C and shaking to remove contaminating debris; the supernatant was discarded. This washing step was repeated twice. The washed tissue pieces were incubated in 40mL pre-warmed HBSS/EDTA (10% FCS, 15mM HEPES, 5mM EDTA in HBSS without Ca²^+^ and Mg²^+^) at 37 °C with continuous shaking for 15 minutes. For small intestinal (SI-LP) and large intestinal lamina propria lymphocytes (LI-LPL) isolation, the tissue pieces were washed in 40mL of Gut Medium (RPMI 1640 supplemented with 15mM HEPES, 5% FCS, 0.2% gentamycin) for 5 minutes at room temperature with gentle shaking to remove residual EDTA. The supernatant was discarded, and the tissue was minced finely on aluminum foil. The minced tissue was divided into two 50mL tubes, each containing 20mL of the medium at 37 °C supplemented with 500µL Liberase TM (0.125mg/mL), 100µL Liberase DH (0.180 mg/mL), and 600µL DNase I (0.180 mg/mL). The digestion mixture was incubated with shaking for 20 minutes at 37 °C. After digestion, the tubes were vortexed, and the contents were filtered through a 190µm mesh into fresh 50mL tubes. The remaining tissue was passed through two additional rounds of digestion under identical conditions. Each digestion step was followed by centrifugation of the flow-through at 453×g for 10 minutes at 4 °C, and the pellets were resuspended in Gut Medium for subsequent filtration. After the final digestion, the suspension was vortexed and filtered through a 190µm mesh into the previous cell-collecting tubes, then diluted with additional Gut Medium. The filtered cell suspension was centrifuged at 453×g for 10 minutes at 4 °C, and the pellet was resuspended in 30mL of HBSS/HEPES buffer. A second centrifugation was performed at 453×g for 10 minutes at room temperature, and the supernatant was discarded. The resuspended pellet (6mL) was layered onto a discontinuous Percoll gradient composed of 6mL of 36% Percoll/Gut medium over 6mL of 67% Percoll/Gut medium. The gradients were centrifuged at 700×g for 30 minutes at room temperature with acceleration set to 3 and deceleration set to 0. Cells located at the interface were collected, diluted with Gut medium, and centrifuged at 453×g for 10 minutes at 4 °C. The final pellet was resuspended in cIMDM for cell counting.

### 2.7 White blood cell count

Numbers and types of leukocytes (lymphocytes, segmented and rod-shaped neutrophils, eosinophils, basophils, and monocytes) were counted and determined. Blood smears were stained with Romanowsky (DiffQuick, Labor + Technik, Eberhard Lehmann GmbH, Germany) according to the manufacturer’s instructions. For each microscope slide 100 cells were counted and classified.

### 2.8 Histology

Tissue samples were stored in buffered formalin solution (Roti-Histofix 4%, Carl Roth GmbH & Co.KG) for 24h at room temperature and protected from light. Thereafter, samples were paraffin-embedded and stained with hematoxylin-eosin and by the Periodic Acid-Schiff reaction (PAS). All measurements were made using the open-source image analysis software QuPath (version 0.5.1).

For tissue eosinophil counts, scoring was as follows: 0, normal (physiological conditions); 1, minimally increased; 2, mildly increased; 3, moderately increased; 4, markedly increased. The scoring was performed by a veterinary pathologist.

For counting the goblet cells, 10 villi and corresponding crypts were randomly chosen from similarly oriented villi and crypts. The area of the villus was measured by outlining its borders with a ruler tool. The goblet cells in each section were counted and divided by the area. The average number of goblet cells per 10 villi (expressed in mm^2^)was used for statistical analysis. All evaluations were performed in a blinded manner.

### 2.9 Flow cytometry

After isolation, the cells were seeded at a quantity of 3 million cells per well in a 96-well plate; lymphocytes were stored at 4° and processed on the next day for the transcription factor staining. For each staining panel Fc receptors were blocked with mouse serum at a concentration of 2µl/ml. For the detection of intranuclear transcription factors, cells were fixed and permeabilized using the FoxP3/Transcription Factor staining buffer set (Thermo Fisher) according to standard protocols. Samples were acquired using BD FACSARIA III and analyzed using Flowjo version 10 (BD Life Sciences, Franklin Lakes, USA). The used antibodies can be found in **Table 1**.

**Table 1:** Antibodies used for flow cytometry.

Target	Fluorochrome	Clone	Isotype	Company
Gd-TCR1	Unlabeled	PGBL22A	IgG1	Kingfisher Biotech
IgG1	APC-Cy7	RMG1-1	IgG1	BioLegend
CD4	PE-Cy7	74-12-4	IgG2b	BD Biosciences
CD3	PerCp-Cy5.5	8C8	IgG2a	BD Biosciences
CD45	Alexa 647	K252.1E4	IgG1	BIO-RAD
Tbet	BV605	4B10	IgG1	BioLegend
Gata3	eFluor 450	TWAJ	IgG2b	ThermoFisher
FoxP3	PE-eFluor 610	FJK-16s	IgG2a	ThermoFisher
Fixable viability dye (DCE)	eFluor 506			ThermoFisher

### 2.10 Gene expression analysis

RNA of frozen tissue samples was extracted for RT-qPCR analysis using the InnuPrep Mini Kit (Analytik Jena, Jena, Germany) according to manufacturer´s instructions. Transcription into cDNA was performed using the High Capacity RNA-to-cDNA kit from Applied Biosystem (ThermoFisher; Waltham, MA, USA) according to manufacturer´s isntructions. Transcribed duplicate DNA probes were amplified and detected using the SYBR-green method (Applied Biosystems, ThermoFisher). Sequences are listed in **Table 2**. All primers were purchased and synthesized from TIB Molbiol (Berlin, Germany).

**Table 2:** Primers for Real-Time qPCR analysis.

*Gene*	F: 5 '- forward primer – 3 '	*Reference*
	R: 5 '- reverse primer – 3 '	
*ARG1* Arginase-1	F: CCAGTCCATGGAGGTCTGTC	(56)
	R: GTGTCTTCCCCAGAGATGGA	
*GAPDH*	F: GGCATGGCCTTCCGTGT	(57)
	R: GCCCAGGATGCCCTTGAG	
*RETNLB* Resistin-Like Beta	F: TCCCTCTGCTCCAAGAAAGA R: CAAGCACAGCCAGTGACAAC	(44)
*IL-13*	F: CTGACCACCAGCATGCAGTACT R: GCTGCAGTCGGAGATGTTGA	(57)
*EPX* Eosinophil Peroxidase	F: TGGCCTCCCAGGGTACAAT R: CAGGAACTTCCTCGCCAAAG	(58)

To calculate the primer efficiencies, a standard curve was created with each primer pair and all samples pooled. mRNA expression was normalized to the housekeeping gene glyceraldehyde-3-phosphate dehydrogenase (GAPDH). The relative gene expression was calculated using the Pfaffl method (59).

### 2.11 Antibody detection by enzyme-linked immunosorbent assay

*A. suum*-specific antibody titers in serum and small intestinal digesta were measured using a sandwich ELISA. At start, 96-well flat-bottom Maxisorp plates (Thermo Fisher Scientific, Waltham, MA, USA) were coated with 20 µg/mL of *A. suum* juvenile E/S overnight at 4 °C. The plates were then washed using a Tecan Hydrospeed microplate washer (Tecan Trading AG, Maennerdorf, Switzerland) and blocked with 200 µL of 3% BSA in PBS for 1 h at room temperature. After blocking, 50 µL of each sample were added to the wells. Internal standards were prepared using pooled serum or intestinal content/digesta samples from infected animals as a positive control. Two-fold serial dilutions of this reference control were performed with starting dilutions of 1:25 for serum and 1:200 for digesta. Test samples were diluted 1:50 for serum, and 1:200 for digesta. Following incubation for 2 hr at room temperature, 50 µL of mouse anti-pig IgA detection antibody (#MCA638GA; Bio-Rad, Hercules, USA) was added and incubated for 1 h. The plates were washed again before adding HRP-conjugated IgG (#STAR117P; Bio-Rad, Hercules, USA) at a 1:10,000 dilution for a further 1 h incubation. After the final wash, 50 µL of TMB substrate solution (Invitrogen, Waltham, MA, USA) was added, and the plates were incubated in the dark at room temperature for 20 min. The enzymatic reaction was stopped by adding 25 µL of 1 M H_2_SO_4_ per well. Absorbance was measured using a Biotek Synergy H1 Hybrid Reader. (BioTek Instruments GmbH, Bad Friedrichshall, Germany). Parasite-specific antibody levels in serum and digesta are expressed as arbitrary ELISA units, calculated following background subtraction of the reference and sample dilutions as recently described (60).

### 2.12 Western blot

Small intestinal tissue samples were lysed in RIPA buffer (Thermo Fisher Scientific, Waltham, MA, USA) for total protein extraction and cOmplete™, Mini, EDTA-free Protease Inhibitor Cocktail tablets (Roche, Sigma-Aldrich/Merck, Darmstadt, Germany). Protein concentrations were determined using a bicinchoninic acid (BCA) assay (Thermo Fisher Scientific), and equal amounts of protein were separated by SDS-PAGE. Proteins were subsequently transferred onto nitrocellulose membranes.

After transfer, membranes were blocked for 2 h in blocking buffer consisting of Tris-buffered saline with Tween-20 (TBST) supplemented with 5% milk powder. Membranes were then incubated with the primary antibody diluted in TBST containing 5% milk powder overnight, followed by incubation with the secondary antibody for 1 h. Protein bands were visualized using an alkaline phosphatase-based substrate solution containing nitro blue tetrazolium and 5-bromo-4-chloro-3-indolyl phosphate substrates (NBT/BCIP; AppliChem, Darmstadt Germany) for chromogenic detection.

For ARG1 detection, a mouse monoclonal anti-Liver Arginase antibody [C5] was used (Abcam, Cambridge, UK; ab239731; 1:500). The secondary antibody was goat anti-mouse IgG H&L conjugated to alkaline phosphatase (Abcam, Cambridge, UK; ab97020; 1:5000). Only the ARG1 band at the expected molecular weight of approximately 35–40 kDa was considered for interpretation.

### 2.13 Statistical analysis

**Figure 1:**
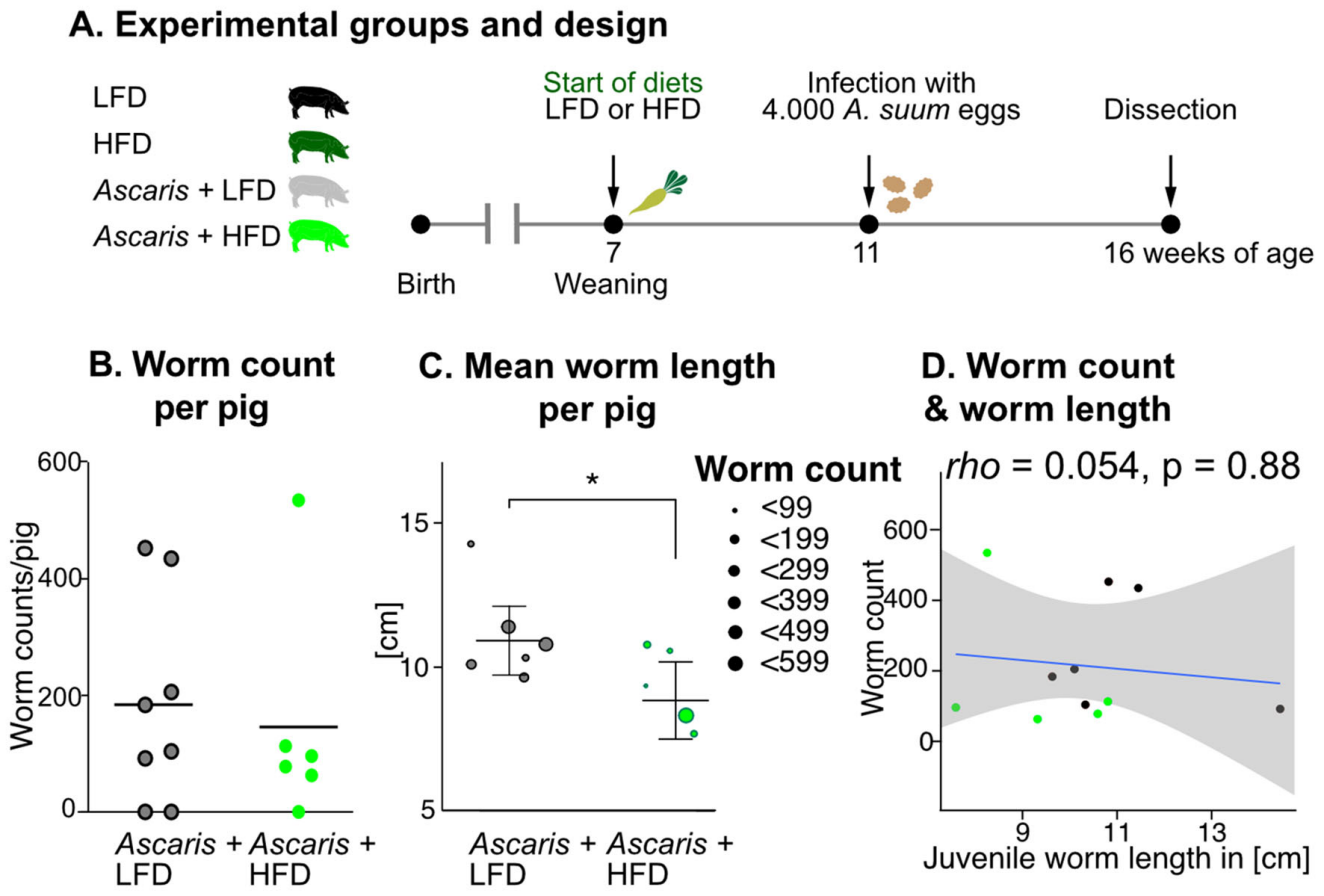
Parasitological parameters. **(A)** Pigs were fed a diet either low or high in fermentable fibers (LFD, HFD) and infected with *Ascaris suum* or kept as naïve controls. Naïve groups comprised four animals each, whereas infected groups consisted of eight animals. Two animals from the “*Ascaris* + HFD” group died due to causes unrelated to experimental settings. **(B)** Intestinal worm burdens as determined on day 35 p.i. **(C)** Mean worm length on day 35 p.i. collected from individual pigs. The size of the dots represents the worm count. To account for unequal worm burdens across individuals in **(C)** a weighted Mann-Whitney-U test was applied. **(D)** A Spearman’s rank correlation analysis reveals no significant relationship between worm counts and worm lengths (*rho* = 0.054, P = 0.88). Because worm count and worm length data were not normally distributed, comparisons were performed using the Mann-Whitney U test. Lines represent means with standard deviation [SD, in **(B)**] and weighted SD of worm length shown in (C) Statistical significance codes *P < 0.05 **(C)**.

**Figure 2:**
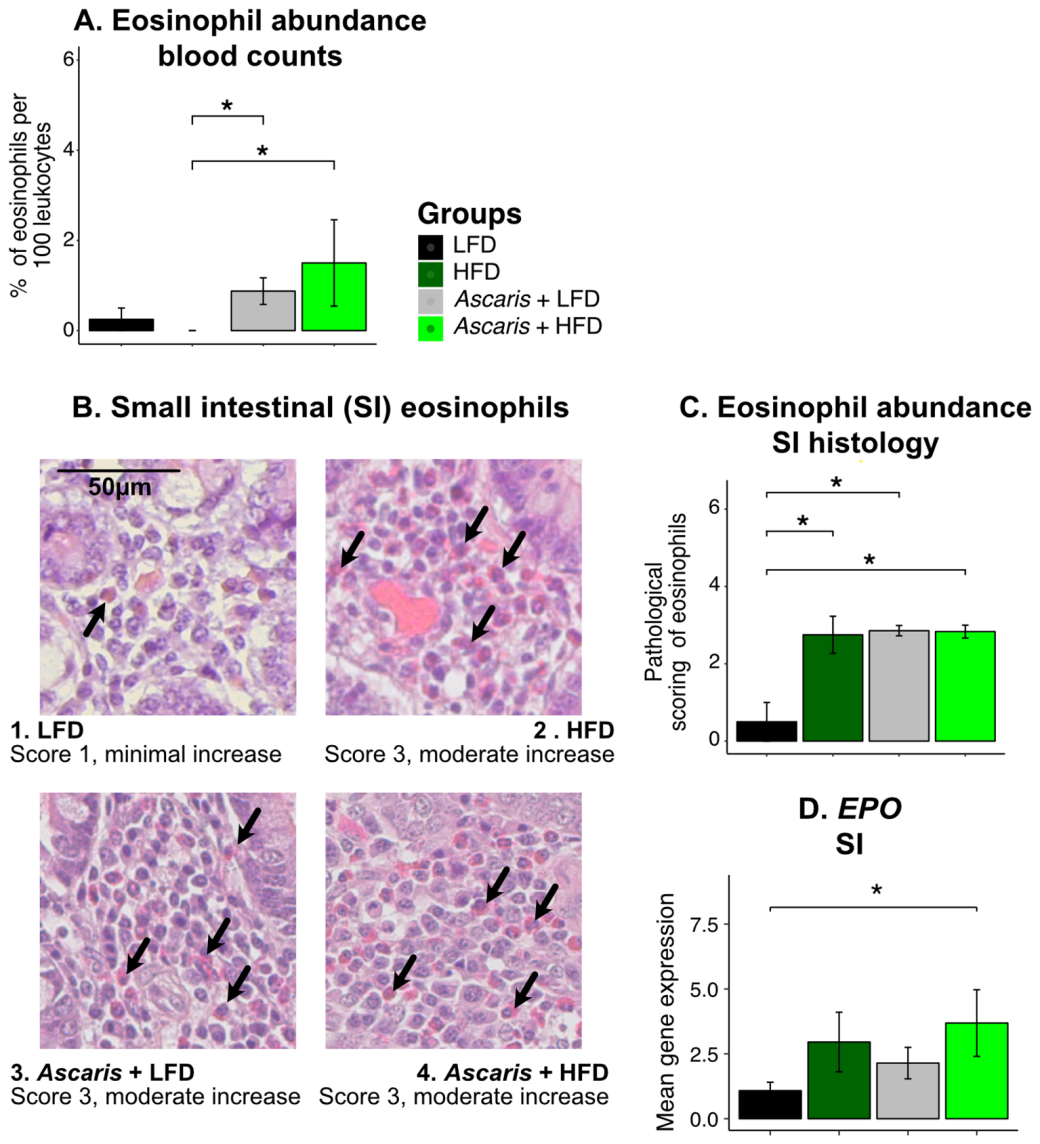
Systemic and mucosal eosinophil responses. **(A)** Relative eosinophil counts in peripheral blood leukocytes collected at day 35 p.i. Data are presented as mean ± SEM. **(B)** Representative pictures of H&E-stained cross section of small intestinal (SI) tissue at day 35 p.i. Arrows point out eosinophils. **(C)** Eosinophil abundance scores determined in SI by histology. Data are presented as mean ± SEM. Scoring ranged from 0 to 4, with 0: physiological condition with few eosinophils present; 1: few and widely scattered eosinophils; 2: mildly increased eosinophil accumulation; 3: moderate increase; and 4: marked increase in eosinophils within the tissue. **(D)** Expression of eosinophil peroxidase (EPO) as determined by qPCR in SI samples at day 35 p.i. Data are presented as mean ± SEM, with significant differences highlighted. Statistical significance codes: *P < 0.05 by Kruskal-Wallis test with Dunn’s *post hoc* test **(A, B)**, and by one-way ANOVA, followed by Tukey’s HSD *post hoc* test **(D)**.

**Figure 3:**
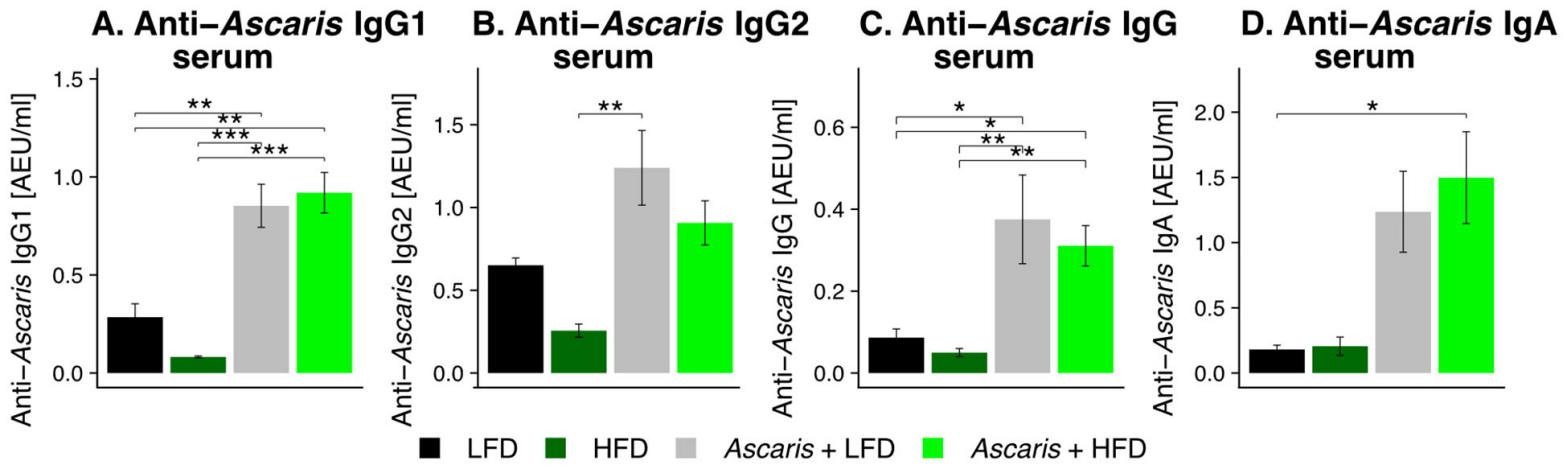
*Ascaris*-specific serum antibody responses. **(A)** *Ascaris*-specific IgG1 levels in serum at day 35 p.i., determined by ELISA. **(B)** *Ascaris*-specific IgG2 levels in serum at day 35 p.i., determined by ELISA. **(C)** *Ascaris*-specific IgG levels in serum at day 35 p.i., determined by ELISA. **(D)** *Ascaris*-specific IgA levels in serum at day 35 p.i., determined by ELISA. Bars represent means ± SEM. Statistical testing was performed by one-way ANOVA followed by Tukey’s HSD *post hoc* test for panels A, B, and D, and by Kruskal-Wallis test followed by Dunn’s *post hoc* test for panel **(C)** Statistical significance is indicated as follows: ***P < 0.001, **P < 0.01, *P < 0.05.

**Figure 4:**
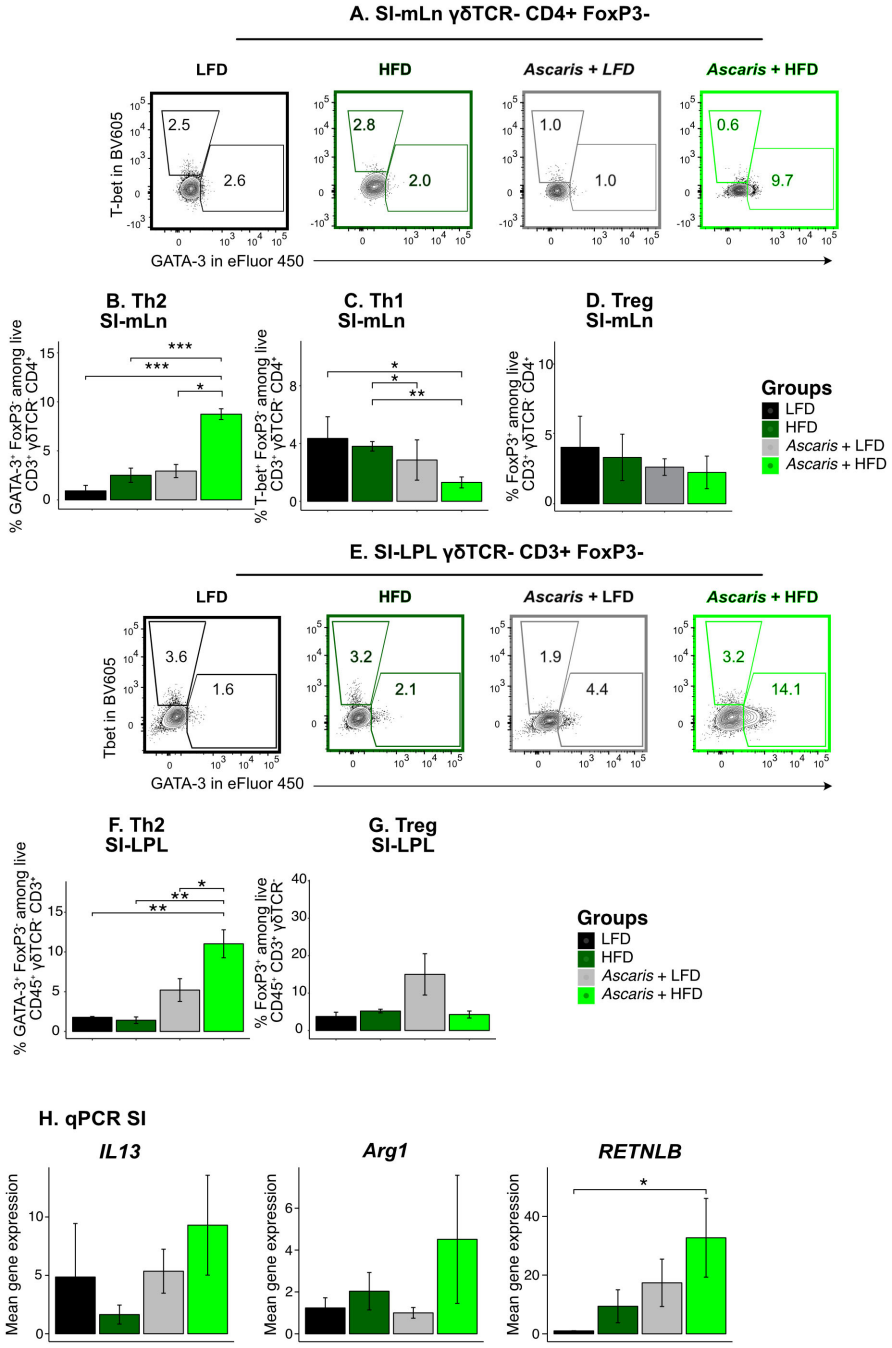
Local Type 2 responses. **(A)** Representative FACS plots depicting GATA-3 and T-bet expression by CD4+ T helper cells isolated from mLn draining the small intestine (SI-mLn). Gating strategy shown in **Supplementary Figure 3** **(A–D)**. Frequencies of GATA-3+ Th2 cells **(B)**, T-bet+ Th1 cells **(C)** and Foxp3+ Treg cells **(D)** among CD3+CD4+γδTCR– lymphocytes in SI-mLn. Supplementary analyzes show the frequencies of the indicated T-cell subsets expressed relative to live cells and, for lamina propria samples, relative to CD45+ leukocytes (**Supplementary Figure 5**). **(D)** Frequencies of FoxP3+Treg cells in SI-mLn. **(E)** Representative FACS plots depicting GATA-3 and T-bet expression by CD3+ T cells isolated from the small intestinal lamina proria (SI-LP). See **Supplementary Figure 4B**. for gating strategy. **(F)** Frequencies of GATA-3+ Th2 cells in the small intestinal lamina propria (SI-LP) among CD45+CD3+γδTCR–FoxP3–. **(G)** Frequencies of FoxP3+ Treg cells among CD45+CD3+ γδTCR– lymphocytes in SI-LP. Th2 and Treg frequencies are reported as % in CD3+ due to poorly resolved CD4 signals in intestinal samples. **(H)** Relative mRNA expression of anti-helminth effector molecules [*IL13* (interleukin-13), *ARG1* (arginase-1), *RETNLB* (resistin-like molecule beta, RELM-β)] in small intestinal tissue. Gene expression values ​​were normalized to the naïve control group. Bars show mean ± SEM. Statistical analysis: Data were first tested for normal distribution and variance homogeneity. For **(B, F, G)**, *P < 0.05 by one-way ANOVA with Tukey’s *post hoc* test. For the other panels **(C, D, H)** non-parametric Kruskal–Wallis test followed by Dunn’s *post hoc* test were applied. Significance codes: ***P < 0.001, **P < 0.01, *P < 0.05.

**Figure 5:**
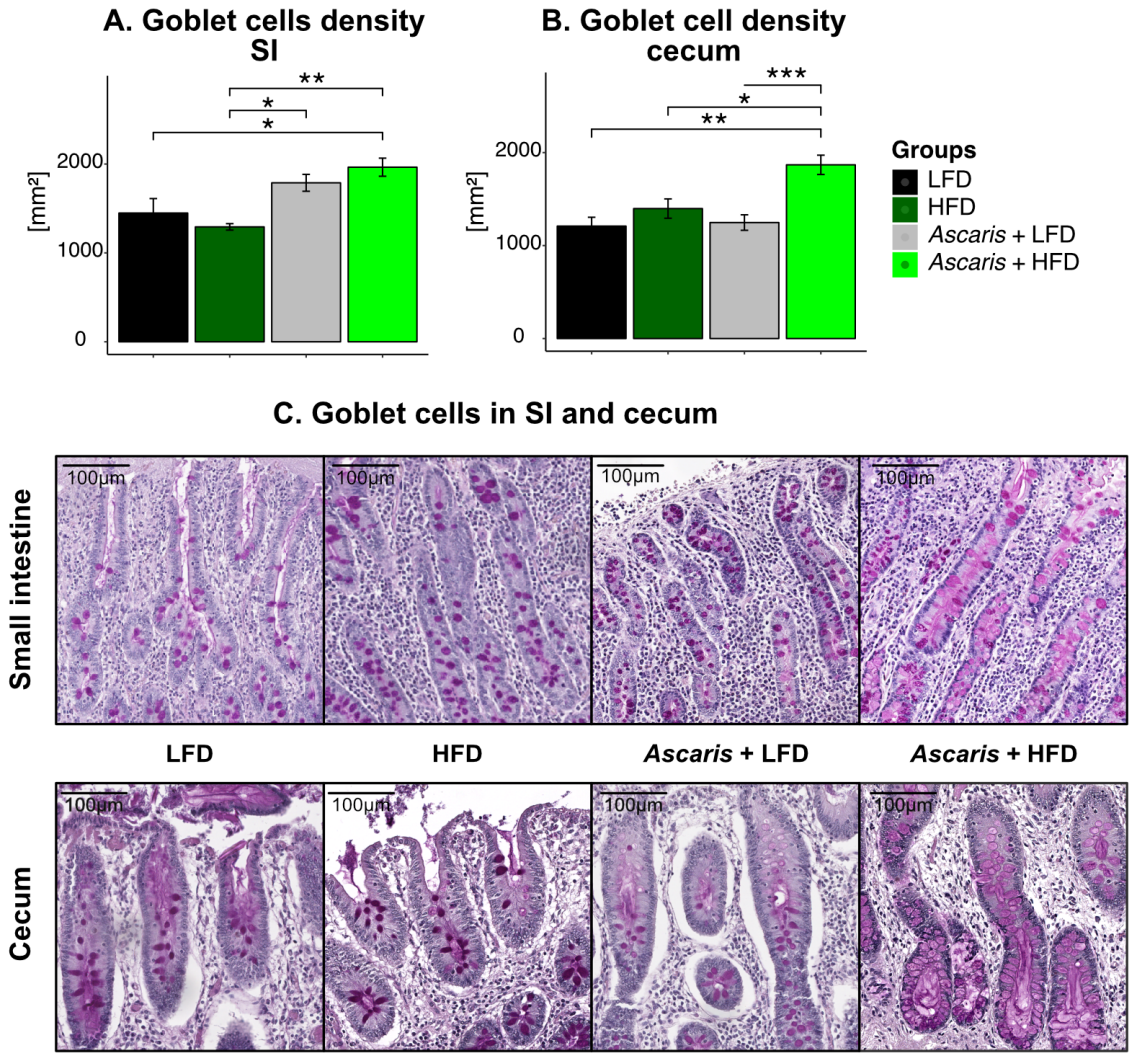
Goblet cell density in small intestine and cecum. **(A, B)** provides representative PAS-stained histological sections of the small intestine **(A)**, and cecum **(B)**, showing goblet cell distribution across experimental groups. The images **(C)** exemplary illustrate differences in goblet cell numbers between the groups. Density of goblet cells per mm^2^. Statistical differences are indicated above the bars, and data are presented as mean ± SEM. The statistical testing was performed by one-way ANOVA with Tukey’s HSD *post hoc* test. Variance homogeneity and normality were confirmed. Statistical significance codes ***P< 0.001, **P < 0.01, *P < 0.05.

Data were analyzed and reported as mean and standard deviation (**Figure 1**) or standard error (SEM, **Figures 2**–**5**). Normality of the data was assessed by performing Shapiro-Wilk-test and the homogeneity of variances was determined by using Levene’s test (rstatix, version 0.7.2) Parameters were analyzed either by one-way ANOVA, followed by Tukey’s HSD *post hoc* test when appropriate. If assumptions of normally distribution of the data were not met data were analyzed using a non-parametric approach with Kruskal-Wallis test and Dunn’s multiple comparison test. For the worm size measurements (length and area), multiple worms were measured per individual pig, resulting in unequal numbers of observations per animal. To account for unequal worm numbers across animals, we applied a dual approach. First, we applied a weighted Mann-Whitney U test in which worm counts were used as the weighting variable. In this rank-based approach, worm length values were ranked with observations representing higher worm numbers contributing proportionally more to the test statistic. This allowed the analysis to account for unequal worm numbers across animals while retaining the full dataset and avoiding arbitrary subsampling.

Second, to assess whether this weighting could bias the comparison due to infection intensity/worm crowding, we tested the relationship between worm burden and worm length. These weighted analyzes were conducted using the sjstats package (version 0.19.1). Second, we performed a correlation analysis to evaluate the potential relationship between worm burden and worm size. Data were analyzed in R Studio (version 09.1–401 for Mac) with the rstatix (version 0.7.2) package (61). Visualization of data was performed using the ggplot2 package (4.0.0).

## 3 Results

### 3.1 Fermentable fiber supplementation reduces *A. suum* juvenile worm size

We aimed to investigate whether fermentable fiber supplementation influences *A.suum* infection in pigs. Weaned female pigs (7 weeks of age) were administered two diets (composition described in **Supplementary Table 1**): a control diet low in fermentable fiber and high in non- fermentable lignocellulose (LFD) versus a diet enriched with inulin and sugar beet pulp, thus high in fermentable fibers (HFD). After four weeks of dietary adaptation, pigs were infected with 4,000 embryonated *A. suum* eggs (shown in **Figure 1A**). At 35 days post infection (dpi), when juvenile *A. suum* worms are back in the small intestine, the HFD group displayed no significant reduction in worm counts but a difference in the distribution, with all pigs except for one having few or no parasites (**Figure 1B**). Interestingly, the “*Ascaris* + HFD” group exhibited a significant reduction in mean worm length (**Figure 1C**) and area (**Supplementary Figure 1**) at 35 dpi. To assess whether worm density influenced individual worm size, a correlation between worm count and mean worm length was examined, but no significant association was detected (*Spearman’s rho* = 0.054, p = 0.88; **Figure 1D**).

Eggs were not detectable at 35 dpi, which forms part of the prepatent stage at this time point. Liver milk spots did not differ between infected dietary groups, and histological evaluation of liver tissue revealed no marked diet-dependent differences in e.g. eosinophilic infiltration (**Supplementary Figure 2**). Lung pathology was surveyed based on perivascular, peri-bronchial, and interstitial inflammation as well as granuloma occurrence, but no marked diet-dependent differences were detected using a semi-quantitative scoring system (62) (**Supplementary Figure 2**).

Assessing the kinetics of type 2 responses in pigs infected with *A. suum*, we have previously shown that eosinophil as well as Th2 accumulation first occurs in the lung (21). Consistent with this, pathological scoring of lung tissues in the present study revealed a significant increase of eosinophils in the infected HFD fed animals. Hence, elevated tissue eosinophil numbers were apparently maintained in the lung tissue of HFD infection group for approximately 25 days after the L3 are expected to have left the organ (**Supplementary Figure 2**). Together, these findings indicate that four-week dietary supplementation with highly fermentable fiber was associated with impaired *A. suum* length, while total worm burden at 35 dpi was not significantly reduced.

### 3.2 Local eosinophil expansion induced independently by *A. suum* infection and fermentable-fiber supplementation

Eosinophils play a pivotal role in helminth infection, exhibiting direct cytotoxic activities via the release of granule proteins, the generation of superoxide anions, and through mediating the humoral immune response (63, 64). Studies in mice demonstrated that an inulin enriched diet induced eosinophilia (65). We therefore determined eosinophil numbers both systemically and locally. Blood smear examinations showed a significant increase in eosinophils in *A. suum*-infected pigs (**Figure 2A**), with an additional trend of an increase in the HFD diet recipients. Pathohistological evaluation of the small intestine revealed a greater eosinophil infiltration in HFD -fed animals, as well as in all infected groups (**Figure 2B**), suggesting that both HFD supplementation and *A. suum* infection are independent but equally strong drivers of eosinophil expansion. Additionally, pulmonary eosinophil scores were increased in HFD-fed infected animals, indicating an enhancement of a local eosinophil proliferation during larval migration (**Supplementary Figure 2**). Gene expression analysis of the eosinophil peroxidase (*EPO*) showed higher levels of eosinophil peroxidase expression in the small intestine of pigs fed the HFD diet and additive effects in the HFD-fed infected group (**Figure 2C**). Therefore, the data suggest a potent influence of the dietary supplementation and the infection on eosinophil numbers and function, potentially leading to a stronger local eosinophil response.

### 3.3 Parasite-specific serum IgG1, IgG2 and IgG increases in infected pigs with no effect related to the diets

*A. suum* infection induced a clear systemic parasite-specific humoral immune response, as reflected by significantly increased serum levels of *A. suum*-specific IgG1, IgG2, total IgG, and IgA in infected pigs compared with non-infected controls (**Figures 3A–D**). These findings confirm robust antigen-specific antibody induction following infection. However, HFD supplementation did not significantly modulate the magnitude of these systemic antibody responses, as no diet-dependent differences were observed within infected animals for IgG1, IgG2, total IgG, or IgA. This suggests that, under the experimental conditions tested, the fermentable fiber intervention did not measurably alter systemic parasite-specific humoral immunity. Nevertheless, *A. suum*-specific IgA showed a trend toward higher levels in HFD-fed infected pigs, indicating a possible diet-associated effect on this antibody isotype that did not reach statistical significance.

### 3.4 Fermentable fiber supplementation strengthens parasite-driven Th2 responses in the small intestinal lymph nodes and intestinal lamina propria

Since we observed a local increase in small intestinal eosinophils following HFD supplementation, we investigated whether this is accompanied by changes in the local adaptive immune response. Analyzes of lymphocyte subsets in the small-intestinal draining mesenteric lymph nodes (SI-mLN), defined as Th2 cells (CD45+CD3+γδTCR-CD4+FoxP3-GATA-3+), Th1 cells (FoxP3- T-bet+), and Treg cells (FoxP3+), as shown in the gating strategy (**Supplementary Figure 4**), showed significantly higher GATA-3+ frequencies in infected HFD fed animals, (**Figures 4A, B**). Th1 (T-bet+) frequencies did not increase with infection but decreased and were significantly lowest in the *Ascaris*-infected and HFD supplemented group (**Figures 4A, C**). In contrast, Treg (FoxP3+) frequencies in the SI-mLN did not differ between groups (**Figure 4D**).

In the small-intestinal and large-intestinal lamina propria (SI-LP, LI-LP), where CD4 detection is less reliable after tissue isolation (66), we analyzed T helper cells defined as viable CD45+CD3+ γδTCR- FoxP3- and categorized the cells by the transcription factor expression (gating in **Supplementary Figure 4**). Under this framework, Th2 (GATA-3+) cells were significantly increased in *A. suum*-infected animals (**Figure 4F**), giving a clearer picture than in the draining lymph nodes. And like in the draining lymph nodes the Th2 cells of SI-LP and the LI-LP were most prominent in the infected and HFD supplemented group (**Figure 4F**, **Supplementary Figure 6B**). Regulatory T cell- (Treg) frequencies significantly increased with the *A. suum* infection clearly detectable in SI-LP, however a reduction of this effect was shown in HFD-supplemented animals (**Figure 4G**). The impact on Treg cells inversely mirrored the GATA-3+ frequencies. Th1 (T-bet+) frequencies remained unchanged across all groups (**Supplementary Figure 6A**). Supplementary ratio analyzes further underline a shift toward GATA3 predominance, particularly in the lamina propria, where the Tbet/GATA3 ratio was reduced in infected HFD-supplemented animals, whereas the same tendency in the mesenteric lymph node did not reach statistical significance (**Supplementary Figure 8**). To complement the frequency-based analyses, we examined fluorescence-intensity-based readouts of GATA3 and FoxP3 including internally normalized expression indices shown in **Supplementary Figure 9**. The expression index was calculated as the ratio of the geometric mean fluorescence intensity (gMFI) of marker-positive cells to that of the corresponding marker-negative cells within the same parent population as inter-day variation in fluorescence intensity can affect comparability across runs (67). The analyzes did not reveal consistent group differences, reflecting increased population proliferation rather than major changes in per-cell transcription factor expression intensity. Overall, these results suggest that *A. suum* infection induces a relative enrichment of Th2 cells that is further enhanced by HFD, accompanied by regulatory T cell frequencies that are reduced in the HFD group, without a corresponding alteration in Th1 cell frequencies.

mRNA expression in the small intestine (**Figure 4H**) was in line with a Th2-skewed immune response. *IL13* expression tended to be elevated following infection independent of the diet. Two genes with a potential function in anthelmintic activity, Arginase-1 (*ARG1*) and Resistin-like molecule β (*RETNLB*) showed a trend toward upregulation in response to HFD and even further by the *Ascaris* infection in HFD fed pigs. In support of the mRNA expression pattern, by Western blotting no ARG1 protein was detectable in small intestinal tissue samples of naïve animals, whereas all HFD fed infected animals showed detectable ARG1 expression (**Figure 4A**, **Supplementary Figure 10**). In contrast, only 1 out of 8 LFD infected pigs exhibited clear ARG1 protein expression, whereas the faint ARG1 protein bands detected in the remaining 7 LFD pigs indicated marginal arginase-1 production in the small intestine of the infection controls.

Collectively, these results indicate that the Th2 response induced by the helminth infection is enhanced by the HFD supplementation. This underscores the potential of fermentable fibers to strengthen the host’s defense mechanisms against *A. suum*. Similar patterns of Th2 immune responses were detectable in the small intestinal draining lymph nodes and in the lamina propria of small and large intestine (**Figure 4**, **Supplementary Figure 6**). As the differences between dietary groups did not consistently reach statistical significance, the infection remains the dominant factor in increasing frequencies of Th2 cells, whereas fermentable fiber exerts a supportive modulatory effect.

### 3.5 Fermentable fiber supplementation and *A. suum* infection synergistically increase goblet cell density

We further determined goblet cell proliferation and hyperplasia, particularly at the site where larvae invade the large intestine. Our data show that at the site where juvenile worms dwell, in the small intestine, *A. suum* infection was the dominant factor driving goblet cell numbers while HFD had no significant synergistic effect (**Figure 5**). At the place where hatched larvae initially invade and where fermentation of the fibers primarily occurs – in the cecum, we observed a pronounced effect on goblet cell density by the HFD diet, which is consistent with the capacity of fermentable fibers to promote goblet cell hyperplasia and mucus production. Thus, goblet cell counts in both the small intestine and cecum reflected the combined impact of infection and diet. In the representative PAS-stained sections, an increase in goblet cell numbers was particularly evident in the infected HFD group (**Figure 5**).

## 4 Discussion

With the high prevalence of *Ascaris suum* infection in European organic pig farms, the rise of anthelmintic resistance in many gastrointestinal nematode species, and with first indications of treatment failure in *Ascaris* infected schoolchildren, there is an urgent need to develop alternative control strategies for roundworm infections (29). Ideally, new strategies allow to reduce infection pressure while reducing the need for the use of anthelmintics. Alternative strategies may thereby not only decelerate the development of parasite drug resistance, but also reduce the environmental sequelae associated with the heavy use of benzimidazoles in farm animals.

The data presented in this study investigate the dietary supply of fermentable fibers as a potential alternative control strategy for *Ascaris* infections. We show that a diet enriched in inulin promotes porcine mucosal eosinophils expansion, Th2 responses, goblet cell expansion and the production of the anti-helminth effector molecule Arg1 and RELM-β accompanied by an impaired worm growth. These findings are coherent with recently published work reporting the accelerated type-2 immune-dependent control of gastrointestinal nematode infection in mice fed an inulin-enriched diet (68). Despite not showing a significant reduction in worm numbers at day 35 p.i. in the present study, the smaller worms and the elevated type 2 responses in inulin fed pigs indicate initiation of host-mediated control. This is consistent with an earlier study which reported significantly reduced *Ascaris* worm burdens in piglets fed inulin-rich chicory roots while exposed to weekly *Ascaris* trickle infection over a period of 7 weeks (46). Consistent with those studies, a limitation of the present experiment is that the infected HFD group was reduced to six animals due to losses unrelated to the experimental treatment. While group sizes in controlled pig infection models are often necessarily smaller than in rodent models, the final group size remains consistent with comparable porcine helminth infection and dietary intervention studies, including studies using six to nine pigs per group (45, 69–71).

In accordance with earlier work from our group (21), *Ascaris* infection alone induced a slowly manifesting local Th2 cell response in the small intestine exceeding baseline levels only after about 5 weeks of *Ascaris* infection. In the current study, the HFD supplementation enhanced this response throughout the gut, most prominently in the lymph nodes and the lamina propria of the small intestine and with responses also assessed in the large intestine where larvae invade. In this compartment, studies assessing type 2 associated defense mechanisms may clarify whether inulin feeding impairs parasite development at very early stages of infection. Previous work has demonstrated that pigs can develop highly effective Th2 responses in the cecum associated with a 99.7% reduction in larval numbers (57). Together with a) the higher resistance of chicory-fed pigs to repeated trickle infection with *A. suum* reported by Jensen and colleagues (46), b) our current finding of the decreased size of juvenile *Ascaris* worms in HFD-fed pigs, and c) the more robust type-2 dependent control of gastrointestinal nematode infection in inulin fed mice (65), these data strongly suggest that fermentable fiber supplementation may significantly support the immune-mediated self-cure and protective immunity against *A. suum* infection in pigs.

Dietary supplementation with fermentable fibers such as inulin confers multiple health benefits, including improved gut motility and intestinal health, a reduced risk of rectal carcinoma and microbial-metabolite driven regulatory T cell expansion in humans, as well as reduced diarrhea incidence and ameliorated intestinal inflammation in pigs (72–75). In pigs, moderate inclusion of dietary fiber rich sources has been associated with lower diarrhea rates and ameliorated intestinal inflammation (76). Although certain dietary fibers can impair nutrient digestibility depending on dose and source (41), they show promising potential to influence early chronic infection and host immunity without compromising pig growth at the supplemented concentration used in the current study (**Supplementary Figure 7**).

While HFD-fed pigs displayed key features of type 2 immune activation in response to *A. suum* infection, the inulin-enriched diet alone was sufficient for strongly increased eosinophil accumulation in the small intestine of naïve HFD pigs. This finding is consistent with earlier reports of diet- as well as infection-driven support of eosinophil accumulation in *A. suum*-infected mice and pigs, and inulin-supplemented mice (57, 63, 68, 77). In mice, inulin feeding in absence of enteric infection was reported to promote the numbers and activity of intestinal and pulmonary group 2 innate lymphoid cells (ILC2), activated by tissue derived IL-33 and associated with increased availability of IL-5 (68). Although intestinal *IL5* gene expression levels were not markedly altered in our study (data not shown), ILC2-derived signals may nevertheless contribute to the extensive accumulation of eosinophils observed in the small intestine of HFD supplemented naïve pigs. Of note, inulin feeding did not induce blood eosinophilia, suggesting that the fiber-rich diet supported the survival of small intestinal eosinophils rather than the mobilization of their precursors from the bone marrow. In contrast, eosinophil mobilization was clearly seen in the blood of the infection groups, irrespective of inulin feeding. Whether prolonged survival of small intestinal eosinophils licenses for more robust anti-*Ascaris* effector responses via release of toxic eosinophil products requires further investigations. Notably, the inulin-induced rise in mouse resistance to infection with *Nippostronglyus brasiliensis*, a nematode migrating to the small intestine via skin and lung, was eosinophil-dependent (68). Furthermore, earlier *in vitro* work showed the antibody-dependent attack of *Ascaris* larvae by porcine as well as human eosinophils (57, 78). Hence, it is well conceivable that enhanced eosinophils allow for more effective control of *Ascaris* infection. Importantly, pulmonary eosinophil accumulation has been demonstrated in inulin-fed mice to result in stronger lung inflammation induced by challenge with the allergenic protease papain (65). Assessing the kinetics of type 2 responses in pigs infected with *A. suum*, we have previously shown that eosinophil as well as Th2 accumulation first occurs in the lung (21). Consistent with this, eosinophil abundance scoring of lung tissues in the present study revealed a significant increase of eosinophils in the infected HFD fed animals (**Supplementary Figure 2**). However, to determine whether an early increase of ILC2/Th2 and eosinophil activity in the lung correlates with the impaired growth of *Ascaris* larval stages, future feeding trials will hence include targeted analyzes of eosinophils performed along the lung stage of *A. suum* infection.

Serum levels of anti-*Ascaris* specific IgG1, IgG2, IgG and IgA did not show any differences between dietary groups but were clearly affected by infection as previously shown by our group (60). The increase in IgG1 is consistent with the type-2-associated immune profile typically induced during helminth infection, whereas elevated IgG2 and total IgG indicate broader parasite-specific antibody class switching and systemic adaptive immune activation. The increase in IgG1 is consistent with a type-2-associated humoral immune profile in pigs, as typically induced during helminth infection. In line with this interpretation, porcine IgG1 has been suggested to reflect type-2-biased humoral responses, whereas IgG2 has been linked to type-1-associated cytokine responses (79, 80). The concomitant increase in IgG2 and total IgG therefore indicates broader parasite-specific antibody class switching and systemic adaptive immune activation. This experimental setup does not allow sufficient resolution to determine whether HFD affects memory B cells or long-lived plasma cells. However, the serum antibody data suggest that systemic *A. suum*-specific antibody titers were less strongly modulated by the dietary regimen than local mucosal immune parameters. Future studies should examine mucosal antibody responses and B cell compartments in greater detail.

Regarding type-2 corresponding cytokines, previous findings at 18 dpi eggs showed increased levels of IL-13 in pigs infected with 2,000 *A. suum* (77). IL-13 is a main type-2 cytokine that can be produced by Th2 cells as well as innate lymphoid cells, including ILC2s. Consistently, our data at 35 dpi show elevated *IL13* expression in *A. suum* infected groups, with no significant differences observed between LFD and HFD diets, interestingly the increase following infection was only seen in HFD not LFD where non-infected animals showed yet higher *IL13* expression. By contrast, HFD diet enhanced gene expression of the anti-helminth effector gene *RETNLB* (RELM-β; resistin like molecule-β) (81, 82), with a similar trend on ARG1 (arginase-1) in the small intestine of infected animals. ARG1 protein detection by western blot further supports the notion that HFD supplementation may strengthen infection-associated type-2 effector mechanisms in the small intestine (**Supplementary Figure 10**). Our data further indicate that fermentable fiber supplementation may amplify, rather than independently induce ARG1 expression during helminth infection. This interpretation is biologically plausible, as ARG1 is linked to alternatively activated macrophage responses, tissue repair, and type-2 immune regulation (83, 84).

Similarly, the *RETNLB* expression pattern suggests that HFD did not simply induce a broad type-2 activation state across all animals, but rather promoted an infection-associated epithelial effector response. *RETNLB* expression was lowest in naïve LFD animals and highest in infected HFD animals, whereas naïve HFD animals and infected LFD animals displayed intermediate levels. Thus, the strongest induction of *RETNLB* occurred when helminth infection coincided with fermentable fiber supplementation. However, exploratory correlation analyzes did not reveal a direct association between small intestinal *RETNLB* expression and parasite growth parameters, as no significant negative correlations were observed with worm thickness, cross-sectional area, or worm length (**Supplementary Figure 3**). This suggests that HFD may enhance this anti-microbial epithelial effector component rather than inducing a uniform increase across all groups.

By contrast, GATA-3+ CD4+ T-cell frequencies in the small intestinal draining lymph node showed a negative association with worm area (Spearman’s rho = -0.58, p = 0.062; **Supplementary Figure 3**) suggesting a possible link between stronger type-2 polarization and reduced parasite growth, although this trend did not reach statistical significance. It may indicate that stronger Th2 polarization at the level of the intestinal draining lymph nodes accompanies impaired parasite development. As draining lymph nodes are involved in the initiation and amplification of adaptive immune responses, this association may reflect enhanced priming or reinforcement of anti-helminth type-2 immunity (85). Together with previous reports of type 2 immune activation during porcine *A. suum* infection (57, 58, 60, 77, 86), the data add new evidence that HFD supplementation may modulate this infection-associated response.

In contrast to studies showing that metabolites derived from the fermentation of dietary fibers promote *FoxP3* expression (75, 87), we observed no increase in intestinal FoxP3^+^ T cell (Treg) frequencies in the HFD supplemented groups. However, *A. suum*-infection alone clearly enhanced the intestinal Treg response. This is in contrast to data from 21 dpi showing no effects on small intestinal FoxP3 expression in *A. suum* infected pigs (88) but in line with general ideas on immune regulation in chronic intestinal helminth infections (89, 90). In our study the Th1/Th2/Treg-balance is shifted toward higher anti-parasitic responses, suppressing regulatory immunity by the type 2 inducing effect of inulin (68).

This pronounced Th2-type response forms part of the classical “weep and sweep response” which involves the activation and hyperplasia of goblet cells to enhance mucus production and facilitate worm expulsion (22, 91). Supplementation with fermentable fibers like inulin and pectin has also been shown to increase goblet cell numbers and stimulate mucin synthesis (92, 93). This is consistent with our observation that the strongest increase in goblet cell numbers occurred in *A. suum* infected animals on HFD fiber diet (**Figure 5**), indicating synergistic effects that may enhance mucus secretion and contribute to impaired worm development.

As mentioned above, dietary fibers can be fermented by the intestinal microbiota into metabolites such as SCFA. However, the causal relationships between diet-associated changes in intestinal microbiome composition, microbial metabolites, host immune responses, and *A. suum* development remain to be determined. Future studies integrating metabolite profiling, microbiome analysis, and functional assays using porcine primary immune cells and *A. suum* life stages will be required to clarify these interactions.

In our study, the main fiber sources were inulin from chicory root and sugar beet pulp. While fermentable fibers are shown to modulate intestinal mucus dynamics (94, 95), evidence for many other fiber types, combinations, and their effects in helminth infection models remain limited. We did not see any growth depression or differences in body weight development between the two dietary groups, suggesting that the experimental diet could be considered from a performance perspective (**Supplementary Figure 7**). However, the fermentable fiber source used represents a defined experimental intervention rather than a cost-optimized feed ingredient. Therefore, its direct translation into commercial pig production may be constrained by economic considerations. Hence, future studies should also evaluate more readily available fermentable fiber sources, such as fruit pomaces or other agricultural by-products, as potential practical application forms.

Ultimately, a fermentable fiber-rich diet could not only offer a promising nutritional strategy to support host immunity and restrict *A. suum* development. This approach may complement conventional deworming programs, especially in organic pig farming, and could help reduce reliance on drugs in livestock where low intake of dietary fiber and ascariasis potentially overlap. A similar overlap also exists in humans and given the close biological similarity between *A. suum* in pigs and *Ascaris* (*A.) lumbricoides* in people (96), these findings may also have translational relevance. Globally, *A. lumbricoides*, is estimated to infect over 700 million people in 2021, with the highest prevalences reported in regions of low- and middle-income countries (97). Concurrently, ongoing transitions of dietary patterns shifting from the traditional high-fiber African-heritage diet towards a more westernized pattern lower in dietary fiber (98) may influence host susceptibility to infection. However, experimental data proving this relationship are lacking. By linking dietary fermentable fibers to helminth control, this study provides a model to explore how nutritional shifts could affect porcine and human Ascariasis.

## Data Availability

The original results presented in the study are included in the article/**Supplementary Material**, further inquiries can be directed to the corresponding author.

## Supplementary material

Supplementary Table 1 Composition of low fermentable fiber diet (LFD) and highly fermentable fiber-supplemented (HFD) diet. The diets were formulated to fulfill age requirements and to be iso-energetic and iso-nitrogenous. ^1^Long-chain inulin, purified from chicory root (Orafti^®^HP, Beneo, Germany). ^2^Arbocel^®^ (containing approximately 65% of lignocellulose, J.Rettenmaier & Söhne GmbH & Co. KG, Rosenberg, Germany) ^3^Calculated according to GfE (99) (ME [MJ/kg] = 0.021503 crude protein + 0.032497 ether extract + 0.016309 starch + 0.014701 organic residue − 0.021071 crude fiber (as analyzed).

Supplementary Figure 1 Mean worm area per individual pig. The panel displays the mean of the worm area calculated for each individual pig in the two infection groups. The size of each dot represents the worm count per individual. A weighted Mann–Whitney U test was applied to account for unequal worm burdens across individuals. *p < 0.05.

Supplementary Figure 2 Liver milk spots, lung pathology and eosinophil abundance. **(A)** Number of liver milk spots determined in Ascaris-infected LFD and HFD fed pigs onday 35 p.i. Data are presented as mean ± SEM. **(B)** Liver eosinophil abundance scores determined by histology. Data are presented as mean ± SEM. Scoring ranged from 0 to 4, with 0: physiological condition with few eosinophils present; 1: few and widely scattered eosinophils; 2: mildly increased eosinophil accumulation; 3: moderate increase; and 4: marked increase in eosinophils within the tissue. **(C)** Lung pathology assessed semi-quantitatively by histology using an adapted cumulative scoring system. The total lung pathology score ranged from 0 to 15 and was calculated as the sum of vascular infiltration, bronchial infiltration, interstitial infiltration (each scored as 0, absent; 1, minimal; 2, mild; 3, moderate; and 4, severe) and granuloma occurrence (0, absent; 1, few/small; 2, several/larger; and 3, numerous and/or necrotic). Data are presented as mean ± SEM. **(D)** Lung eosinophil abundance scores determined by histology a described in **(B)**. Data are presented as mean ± SEM. Statistical significance codes: *p < 0.05, **p < 0.01 by Wilcoxon rank-sum test **(A)** and Kruskal-Wallis test with Dunn’s *post hoc* test **(B-D)**.

Supplementary Figure 3 Correlation analyses between Ascaris mean area and effector responses. **(A)** Worm growth expressed as mean parasite surface area does not correlate with relative RETNLB expression and worm area in [cm²] (ρ = −0.24, P = 0.51). **(B)** Correlation between the frequency of SI-mLn GATA-3^+^ FoxP3^-^ cells among live CD3^+^ γδTCR^-^ CD4^+^ T cells and worm area in [cm²] (ρ = −0.58, P = 0.062). Data points are color-coded by experimental group. Lines indicate correlation trends. Correlations were assessed by Spearman correlation analysis.

Supplementary Figure 4 Gating strategy of Th2, Th1 and Treg cells in small intestinal mesenteric lymph nodes (SI-mLn) and small intestinal lamina propria lymphocytes (SI-LP). Si-mLN **(A)** and SI-LP **(B)** cells were gated based on excluding doublet cells using FSC-A vs. SSC-A (not shown). The gating strategy includes gating on live cells, CD45^+^ (only in SI-LP), followed by gating on CD3^+^, CD4^+^ only in (SI-mLn), γδTCR^-^, CD4^+^. For determining regulatory T cells gating included FoxP3^+^cells. Further gating on FoxP3^–^GATA-3^+^ cells was performed for identifying Th2 helper cells and FoxP3^–^T-bet^+^ cells for identifying Th1 cells. GATA-3 positivity was determined based on the γδTCR^+^ fraction, as this subset shows high frequencies of GATA-3 (100) and clearly displays two distinguishable populations [**(A)** in gray, not shown for **(B)**].

Supplementary Figure 5 Frequencies of CD4+ T cell subsets expressed as percentage of live and live CD45+ cells. **(A)** Frequencies of Th2, Th1 and Treg cells within live MLN cells. **(B)**. Frequencies of the corresponding subsets in live CD45+ leukocytes derived from small intestinal LP. Bars represent mean values ​​and error bars indicate SEM. Groups are shown as LFD, HFD, Ascaris + LFD, and Ascaris + HFD. Statistical significance was assessed using Kruskal-Wallis tests followed by pairwise Wilcoxon tests for panels **(A-D)**, and by one-way ANOVA followed by Tukey’s *post hoc* test for panels **(E, F)**. Horizontal brackets indicate significant pairwise differences, with asterisks denoting adjusted P values ​​(P < 0.05, *P < 0.01, **P < 0.001).

Supplementary Figure 6 Frequencies of Th1 and Th2 cells in intestinal lamina propria. **(A)** Frequencies of T-bet+ Th1 cells in CD3+ cells derived from the small intestinal lamina propria. **(B)** GATA-3+ Th2 cells in CD3+ cells derived from the large intestinal lamina propria (LI-LPL). Statistical significance was assessed using one-way ANOVA followed by Tukey’s post hoc test. Brackets indicate significant pairwise differences, with asterisks denoting adjusted P values (***P< 0.001, **P < 0.01, *P < 0.05).

Supplementary Figure 7 Body weight development. Average body weight in kilograms. Each data point represents the mean body weight at a given time, with vertical lines indicating the error of the mean (SEM).

Supplementary Figure 8 Ratio of Th1 to Th2 cells in small intestinal mLN and LPL. **(A)** Th1/Th2 ratio in live MLN cells according to the frequencies of T-bet+ and GATA-3+ T cells. **(B)** Th1/Th2 ratio in live CD45+ cells from the small intestinal lamina propria. Bars represent means ± SEM. Groups are shown as LFD, HFD, Ascaris + LFD, and Ascaris + HFD. Statistical significance was assessed using Kruskal-Wallis tests followed by pairwise Wilcoxon tests. Horizontal brackets indicate significant pairwise differences, with asterisks denoting adjusted P values ​​(*P < 0.05).

Supplementary Figure 9 Estimation of GATA-3 and FoxP3 protein expression in Th2 cells. The GATA-3 and FoxP3 protein expression index was determined according to the geometric mean fluorescence intensity (gMFI) obtained for the GATA-3 signal in the GATA-3+ gate (Th2) divided by the gMFI of the cells in the GATA-3- gate (other T cells). **(A)** GATA-3 and **(B)** FoxP3 protein expression index in small intestinal MLN and **(C, D)** in small intestinal lamina propria. No statistical significances were seen using Kruskal-Wallis tests followed by pairwise Wilcoxon tests.

Supplementary Figure 10 Representative Western blot analysis of Arg-1 protein expression in small intestinal samples from naïve pigs fed LFD or HFD (upper picture, apart from band Ascaris + LFD) and from Ascaris infected pigs fed the different diets (lower picture). Liver samples from pigs and mice were included as positive controls. The boxed region marks the expected Arg-1 signal.
