# Supplementary material for: Fermentable fiber supplementation in pigs promotes anti-parasitic defense mechanisms and impacts parasite growth in Ascaris suum infection
Source: Front Immunol. 2026 Jul 22;17:1788681. doi: 10.3389/fimmu.2026.1788681 (PMC13437497; doi:10.3389/fimmu.2026.1788681)
Supplement: Supplementary file 1 [file Table1.docx]

Supplementary Material

**Fermentable fiber supplementation in pigs promotes anti-parasitic defense mechanisms and impacts parasite growth in *Ascaris suum* infection**

**Philipp Höfler, Josephine Schlosser-Brandenburg, Arkadi Kundik, Sebastian Rausch, Eva-Maria Saliu, Larissa Oser, Anja Kühl, Robert Mugo, Alexandra Laubschat, Zaneta Musimbi Kidiavai, Ankur Midha, Robert Klopfleisch, Jürgen Zentek, and Susanne Hartmann***

*** Correspondence:** Corresponding Author: Susanne.Hartmann@fu-berlin.de

**Supplementary table 1:
Composition of low fermentable fiber diet (LFD) and highly fermentable fiber-supplemented (HFD) diet.** The diets were formulated to fulfill age requirements and to be iso-energetic and iso-nitrogenous.

| **Ingredient (g/kg)** | **Starter diet** | | **Grower diet** | |
| --- | --- | --- | --- | --- |
|  | **LFD I** | **HFD I** | **LFD II** | **HFD II** |
| Dried sugar beet pulp | – | 50 | – | 80 |
| Inulin^1^ (from chicory root) | – | 50 | – | 50 |
| Corn | 411 | 340 | 420 | 374 |
| Wheat | 200 | 223 | 234 | 230 |
| Soybean Meal | 138 | 136 | 190 | 180 |
| Skimmed milk powder | 100 | 100 | – | – |
| Soy oil | 50 | 45 | 40 | 30 |
| Lignocellulose^2^ | 35 | - | 50 | – |
| Monocalcium phosphate | 15 | 15 | 15 | 15 |
| Mineral vitamin mix | 12 | 12 | 12 | 12 |
| Calcium carbonate | 10 | 8 | 8 | 8 |
| Tixosil | 8 | – | 10 | – |
| Lysine | 8 | 8 | 8 | 8 |
| Threonine | 7 | 7 | 7 | 7 |
| Methionine | 5 | 5 | 5 | 5 |
| Tryptophan | 2 | 2 | 2 | 2 |
| **Analysed nutrient content** |  |  |  |  |
| Metabolisable energy^3^ (MJ/kg) | 13.7 | 13.8 | 13.2 | 13.6 |
| Dry matter (g/kg) | 909 | 910 | 913 | 914 |
| Crude ash (g/kg DM) | 54.7 | 55.1 | 52.3 | 50.3 |
| Crude protein (g/kg DM) | 193 | 182 | 184 | 185 |
| Crude fat (g/kg DM) | 70.0 | 64.3 | 61.7 | 46.0 |
| Crude fiber (g/kg DM) | 61.4 | 50.4 | 75.0 | 52.8 |
| Starch (g/kg DM) | 426 | 485 | 445 | 457 |
| Calcium (g/kg DM) | 7.8 | 9.0 | 7.1 | 7.5 |
| Phosphorus (g/kg DM) | 7.3 | 7.1 | 6.8 | 6.4 |

^1^Long-chain inulin, purified from chicory root (Orafti®HP, Beneo, Germany).

^2^Arbocel® (containing approximately 65% of lignocellulose, J.Rettenmaier & Söhne GmbH & Co. KG, Rosenberg, Germany)

^3^Calculated according to GfE (100) (ME [MJ/kg] = 0.021503 crude protein + 0.032497 ether extract + 0.016309 starch + 0.014701 organic residue − 0.021071 crude fiber (as analyzed).
